# Opisthenar microvessel area as a sensitive predictive index of arterial stiffness in hypertensive patients

**DOI:** 10.1038/s41598-021-02294-z

**Published:** 2021-12-08

**Authors:** Zhen Yi Guo, Chen Chen, Xin Jin, Zai Hao Zhao, Lan Cui, Yin Hua Zhang

**Affiliations:** 1 Yanbian University Hospital, Yanji City, Jilin Province China; 2 Department of Physiology and Biomedical Sciences, Ischemic/Hypoxic Disease Institute, Seoul National University, College of Medicine, Seoul, Korea; 3 Division of Cardiovascular Institute, University of Manchester, Manchester, UK

**Keywords:** Physiology, Medical research

## Abstract

We aimed to analyze whether opisthenar microvessel area (OMA, measured with Optical Coherence Tomography (OCT) angiography) was associated with blood pressure (BP), arterial stiffness and whether OMA can predict arterial stiffness in hypertensive (HTN) patients. Results from 90 participants showed that BP, brachial-ankle pulse wave velocity (baPWV) and ankle brachial index (ABI) were significantly higher but OMA (in control, with cold- and warm-stimulation, NT, CST, HST and the differences, CSD, HSD) were significantly reduced in HTN group (n = 36) compared to non-HTN (n = 54). NT, CST, HST and HSD showed negative correlations with baPWV and ABI in all participants, female (n = 47) and male group (n = 43), but the correlation was absent when the participants were divided into HTN and non-HTN. Logistic Regression analysis showed that only baPWV was a significant risk factor for HSD (OR 19.7, 95%CI 4.959–78.733, *p* < 0.0001) but not the age, BMI, smoking, drinking or exercise status (*p* > 0.05). Receiver Operating Characteristics analysis for HSD was 0.781, 0.804, 0.770, respectively. HSD < 9439.5 μm^2^ predicted high BP and arterial stiffness (95% CI in all participants: baPWV, 0.681–0.881, SBP, 0.709–0.900, DBP, 0.672–0.867, *p* < 0.001). These results suggest that OMA is a sensitive index to predict arterial stiffness in HTN population.

## Introduction

Hypertension (HTN) is the main risk factor of fatal cardiovascular diseases due to the comorbidities in vasculature including myocardial infarction, atherosclerosis, cerebral hemorrhage, stroke, diabetes and renal failure^1–3^. The hypertensive population is estimated to be over 10% worldwide, and the numbers are on the rise with the new diagnostic criteria (systolic and diastolic blood pressure > 130/80 mmHg)^4^. Early diagnosis and management of HTN to reduce vascular dysfunction are of vital importance.

Chronic HTN is associated with arterial remodeling and dysfunction—e.g., aortic dissection and aneurysm, arterial calcification and atherosclerosis^2,5,6^. General consensus is that arterial stiffness of aorta and large conduit arteries are independent risk factors of cardiovascular mortality and morbidity, especially in aged population with HTN. Sustained blood pressure elevation and arterial stiffness increase vascular pulsatility, if left untreated, result in endothelial injury and peripheral arterial diseases. Consequently, reduced blood flow causes distal arterial narrowing or closure of the circulatory lumen, a phenomenon called microvascular rarefaction (MR)^7,8^. Concomitant stimulation of sympathetic nervous system, renin-angiotensin- aldosterone system and endothelin system as well as systemic or local inflammation in HTN exacerbate the blood flow reduction through impaired microcirculation and causes vital organ damage^2,8,9^. Accordingly, changes in microcirculation reflects arterial dysfunction, which can be used to predict cardiovascular disease-oriented organ function.

Recently, optical coherence tomographic (OCT) angiography is recognized to be a useful tool to visualize the microcirculation non-invasively and analyze the association with various diseases^10,11^. E.g., choriocapillaris flow deficit, measured using OCT, has been associated with high blood pressure and kidney dysfunction in HTN patients^12^. Furthermore, OCT images have shown that retinal capillary densities were reduced in diabetic and HTN patients^13,14^. Conversely, carotid plasticity and stent treatment reversed capillary density^15^. In line with these findings, retinal capillary remodeling has been shown to correlate with arterial stiffness and kidney dysfunction^16^. These compelling evidences indicate that OCT angiography is a reliable tool to detect the microcirculation changes and MR for cardiovascular monitoring under various disease conditions. Very recently, high-resolution images of cutaneous vessels are measured with OCT angiography and the vessel densities or diameters are shown to respond well to physiological stimulus (e.g. warm) in both healthy and diseased populations^17–19^.

The associations between cutaneous microvessels and arterial dysfunction parameters in HTN patients are unidentified. Accordingly, we aimed to investigate whether opisthenar microcirculation was associated with arterial dysfunctions in HTN patients and whether opisthenar microvascular changes to physiological stimuli (cold or warm stimulus) could be a useful index in predicting the pathology in this group of patients. We measured the opisthenar microvessel area (OMA) using OCT angiography and baPWV and ABI with non-invasive automatic device in 90 volunteers with and without HTN to validate the clinical application of this parameter.

## Materials and methods

### Study design and participants

90 volunteers are recruited from the Physical Examination Center of Yanbian Affiliated Hospital in China Between September 2020 and January 2021. Individuals with medical history of cardiovascular diseases, such as heart failure, stroke, diabetes (including those with high fasting glucose in physical examinations within 6 months), kidney diseases and chronic or acute infection were excluded from the study. All individuals are at fasting state on the day of OCT imaging and brachial-ankle pulse wave velocity (baPWV), ankle-brachel index (AMI) measurements and no medication were taken for the previous 24 h. Examiners are divided into HTN (systolic blood pressure ≥ 140 mmHg, diastolic blood pressure ≥ 90 mmHg, n = 36,) and non-HTN group (systolic blood pressure < 140 mmHg, diastolic blood pressure < 90 mmHg, n = 54). 26 HTN participants take nifedipine (30 mg/once/day) regularly. 10 did not take antihypertensive medication before. The study was conducted in accordance with the Declaration of Helsinki, and the protocol was approved by the Ethics Committee of the Affiliated Hospital of Yanbian University. The informed consent was obtained from all participating subjects.

### Brachial ankle pulse wave velocity (baPWV) and ankle-brachial index (ABI) measurement

baPWV and ABI were measured using an automated device (HBP-8000, Omron, Japan) as described elsewhere^20^. Brachial and ankle wristbands were placed in supine position and two consecutive measurements were conducted after relaxed for 5 min. Data processing is followed straight after the second measurement. baPWV and ABI of both left and right values of the second measurement were analyzed when the readings were similar (or third measurement was taken if inconsistent results were recognized).

### Optical coherence tomography (OCT) angiography measurement

**Figure 1:**
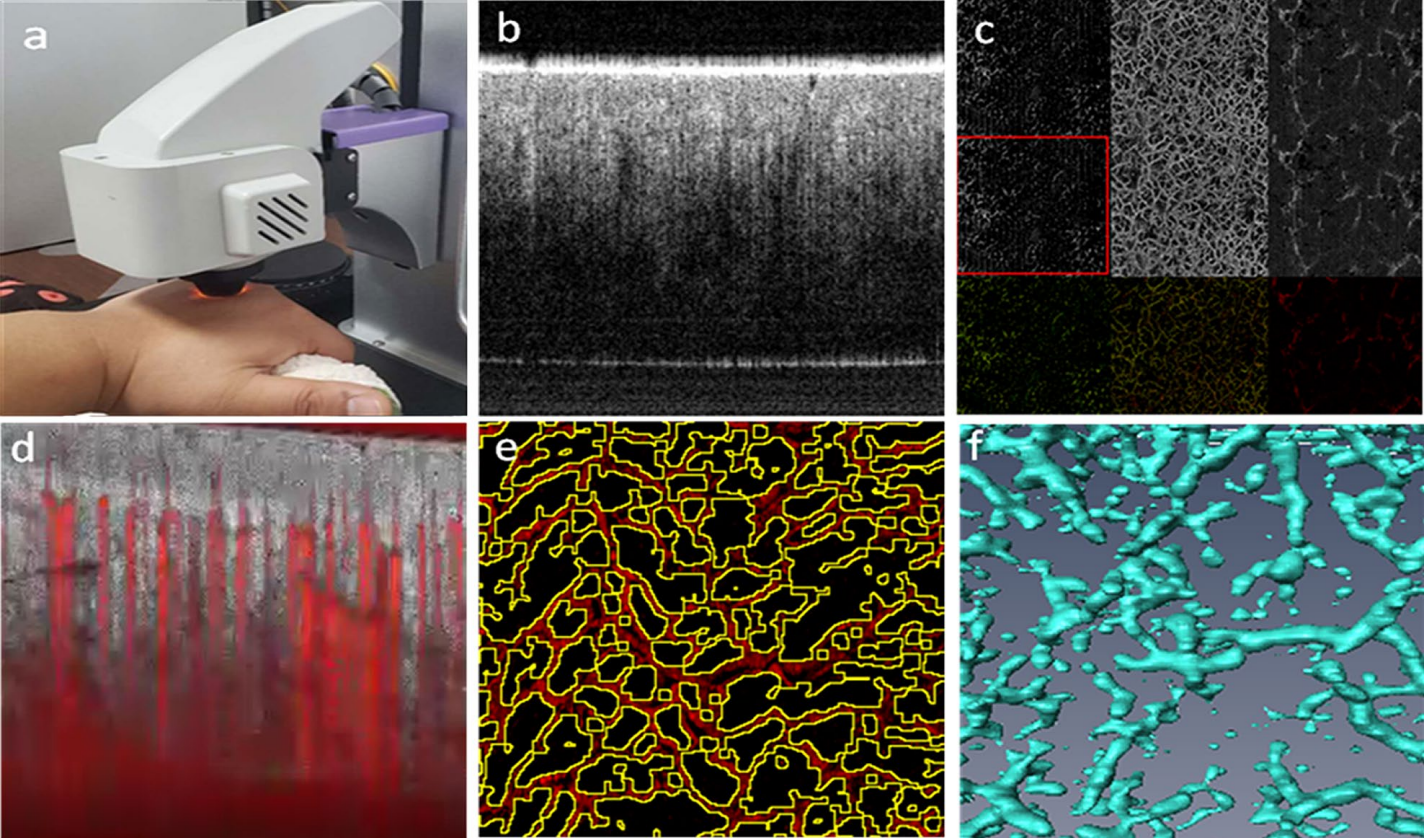
(**a**) Probe position; (**b**) acquisition software main interface; (**c**) blood vessel imaging window; (**d**) blood flow video; (**e**) computer connection diagram; (**f**) three-dimensional imaging diagram.

All 90 participants were examined on the back of hand (1 cm from the middle finger bone) under the detecting probe using a non-invasive imaging system (OCT model, Micro-VCC, UK, Fig. 1a) as described elsewhere^21^. Briefly, a high-speed super-luminescent diode (central wavelength: l060 nm, repetition rate: 100 kHz, spectral bandwidth: 100 nm) scanned the field of view (4 mm × 4 mm) with 16 μm/pixel resolution. Laser reflectance of the surface of flowing red blood cells depict microvessels under the skin. Longitudinal (x-axis) and cross-sectional area (y axis) were scanned and data were processed for quantitative measurement and vessel density. To obtain 3-dimentional images, 250 A-lines through B scan (x-axis) and 250 B-scan (y-axis) with four repeats forming a cube of approximately 4 mm (x axis) × 4 mm (y axis) × 3.8 mm (z axis) for data processing. The structure, shape and branch of the blood vessels were analyzed in the built-in analysis software (Angiotool).

First, observe the blood vessels in the upper layer of epidermal structure, small arteries, venules and capillaries generated color blood vessel diagram (Fig. 1b,c). Blood flow signal diagram could be observed from the skin surface to the middle and bottom layer indicating the dynamic flow (Fig. 1d). The middle to bottom layer data were constructed to blood vessel conjunction images (Fig. 1e) and the blood vessels in the measured area were calculated automatically in the software. The parameters such as surface area of the field of view, blood vessel area, percentage of total blood vessel area, total number of connections, joint density, total blood vessel length, average blood vessel length, total number of end points were provided in the system. 3D microvessel area were formed in the built-in 3D imaging software (Fig. 1f).

Each individual underwent examination in three stages. After the measurement at normal room temperature (NT at ~ 22 °C, 1st stage), the hand was covered by a medical cold pad (Lan Luo 20180028, Shanghai Dianyu Trading. Co. Ltd, − 8 ± 2 °C) for 45 s and measured the same area after 1 min (the 2nd stage). Rest 30 min for the normalization, the hand was covered by a medical hot pad (Lan Luo 20180014, Shanghai Dianyu Trading. Co. Ltd, 42 °C ± 2) for 45 s, took the measurement in the same area. The hand was kept still during the test.

### Statistical analysis

SPSS 19.0 (IBM, Armonk, NY, USA) statistical software was used for data analysis. The data with the normal distribution was expressed as the mean ± standard deviation (mean ± SD). Non-parametric statistical analysis was used for comparison between groups. The Spearman correlation analysis was used to analyze the correlation of each test parameter, and the receiver operating characteristic curve was used to explore the relationship between the arterial stiffness and OMA. Logistic Regression analysis was used to study the correlations between related risk factors (age, BMI, smoking history, drinking history, exercise habits, arteriosclerosis index) and HSD, and findings were reported with the beta coefficient (β), SE, OR 95% CI. *p* < 0.05 was considered to be statistically significant.

## Result

A total of 90 examiners were divided into normal blood pressure group (Non-HTN) and hypertension group (HTN) according to the day of blood pressure measurement and their HTN history. Individuals with complete data sets were included in the analysis. Age, body mass index, gender, systolic blood pressure, diastolic blood pressure, and mean blood pressure were different between two groups (Table 1, *p* < 0.0001). The brachial-ankle pulse wave velocity (baPWV) and ankle-brachial index (ABI) of both left and right side were significantly higher in HTN (Table 1, *p* < 0.0001).

**Table 1:** Descriptive statistical analysis among general information, blood pressure, and arterial stiffness.

Variable	All n = 90	Non-HTN n = 54	HTN n = 36	*p*
**General information**				
Age (year)	46.19 ± 13.31	41.61 ± 14.5	54.16 ± 5.92	0.000**
BMI (kg/m^2^)	24.44 ± 4.10	23.59 ± 4.53	25.72 ± 2.97	0.003*
Gender (male/female)	43/47	18/36	25/11	0.001*
Smoking history	29	8	21	
Drinking history	33	9	24	
Exercise habits	11	7	4	
**Blood pressure**				
SBP (90–140 mmHg)	131.7 ± 24.88	115.25 ± 15.03	156.36 ± 13.76	0.000**
DBP (60–90 mmHg)	83.74 ± 14.85	75.74 ± 11.59	95.75 ± 10.51	0.000**
MAP (70–105 mmHg)	99.87 ± 16.63	89.2 ± 9.94	115.88 ± 10.68	0.000**
**Arterial stiffness**				
baPWV.R (cm/s)	1443 ± 488	1134 ± 195	1906 ± 425	0.000**
baPWV.L (cm/s)	1462 ± 459	1157 ± 142	1920 ± 383	0.000**
ABI.R	1.08 ± 0.1	1.04 ± 0.07	1.16 ± 0.08	0.000**
ABI.L	1.09 ± 0.1	1.05 ± 0.08	1.15 ± 0.11	0.000**

**Figure 2:**
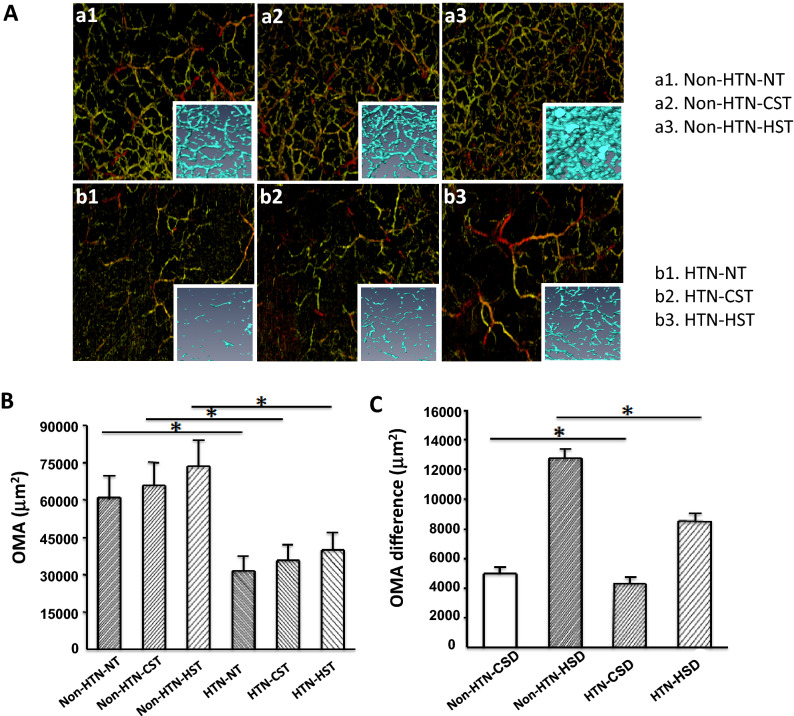
(**A**) Microvascular network imaging under the back of the hand, three-dimensional blood vessel model imaging, a1 normal skin temperature test (NT), a2 cold stimulation test (CST), a3 warm stimulation test (HST), b1 HTN in NT, b2 HTN in CST, b3 HTN in HST; (**B**) histogram of variance of blood vessel area; (**C**) histogram of variance of blood vessel area; non-HTN n = 54, HTN n = 36, * indicates that the comparison between NT, CST, HST, CSD, HSD in non-HTN vs. NT, CST, HST, CSD, HSD in HTN, *p* < 0.01.

OCT angiographic data of opisthenar microvessel and averaged microvascular area (OMA) from non-HTN and HTN individuals before and after cold and warm-stimulation (NT, CST, HST) were shown in Fig. 2. The area and density of blood vessels in the examined field were greater in non-HTN (Fig. 2A,B). 3D images showed significantly greater microvessel area in non-HTN (Fig. 2Aa1–b1 inset), indicating greater blood flow. Both cold- and warm-stimulation increased blood vessel area in non-HTN and in HTN, with higher responses shown with warm-stimulation in both groups (Fig. 2A,B). Re-established 3D model showed significant expansion of the microvessels in non-HTN compared to those in HTN (Fig. 2Aa2,3–b2,3 inset), suggesting greater changes in non-HTN. Indeed, after cold and warm-stimulation, the changes of microvessel density (CSD, HSD) was significantly greater in non-HTN compared to those in HTN (Fig. 2C). These results suggest that microvascular density and blood flow in OMA were reduced in HTN and their response to stimulation was attenuated.

Next, we examined the correlations between baPWV or ABI and OMA in non-HTN and HTN groups. In all 90 examiners, baPWV and ABI (both left and right) were negatively correlated with OMA-NT, CST, HST (Table 2-All). Between CSD and HSD, only HSD were negatively correlated with baPWV or ABI, CSD did not show any correlation (Table 2-All). Therefore, the higher arterial stiffness indexes, the smaller microvascular area. Microvascular response to warm stimulus is inversely associated with arterial dysfunction. However, no correlations were observed between OMA and baPWV or ABI, when the groups were separated into non-HTN and HTN (Table 2-non-HTN and HTN).

**Table 2:** Correlation analysis between microvessel area and arterial stiffness in normal population and hypertensive population.

Variable	ALL n = 90		Non-HTN n = 54		HTN n = 36	
	r	*p*	r	*P*	r	*p*
**baPWV.L**						
NT	− 0.806	0.000**	0.221	0.108	− 0.132	0.443
CST	− 0.801	0.000**	0.008	0.952	0.081	0.638
HST	− 0.808	0.000**	− 0.095	0.494	− 0.052	0.764
CSD	− 0.054	0.616	− 0.160	0.247	0.225	0.188
HSD	− 0.403	0.000**	− 0.208	0.131	0.074	0.667
**baPWV.R**						
NT	− 0.775	0.000**	− 0.059	0.670	− 0.176	0.305
CST	− 0.771	0.000**	− 0.177	0.201	0.069	0.687
HST	− 0.773	0.000**	− 0.087	0.532	− 0.106	0.539
CSD	− 0.052	0.624	− 0.192	0.165	0.264	0.119
HSD	− 0.374	0.000**	− 0.109	0.434	0.064	0.711
**ABI.L**						
NT	− 0.415	0.000**	0.246	0.073	0.014	0.935
CST	− 0.420	0.000**	0.165	0.235	0.154	0.369
HST	− 0.425	0.000**	0.108	0.438	0.068	0.695
CSD	− 0.067	0.531	− 0.124	0.370	0.124	0.470
HSD	− 0.245	0.020*	− 0.104	0.454	0.052	0.764
**ABI.R**						
NT	− 0.583	0.000**	0.056	0.689	− 0.161	0.348
CST	− 0.589	0.000**	− 0.088	0.528	− 0.082	0.634
HST	− 0.559	0.000**	0.044	0.754	0.147	0.392
CSD	− 0.085	0.424	− 0.115	0.408	0.109	0.527
HSD	− 0.199	0.060	0.006	0.968	0.293	0.083

**Figure 3:**
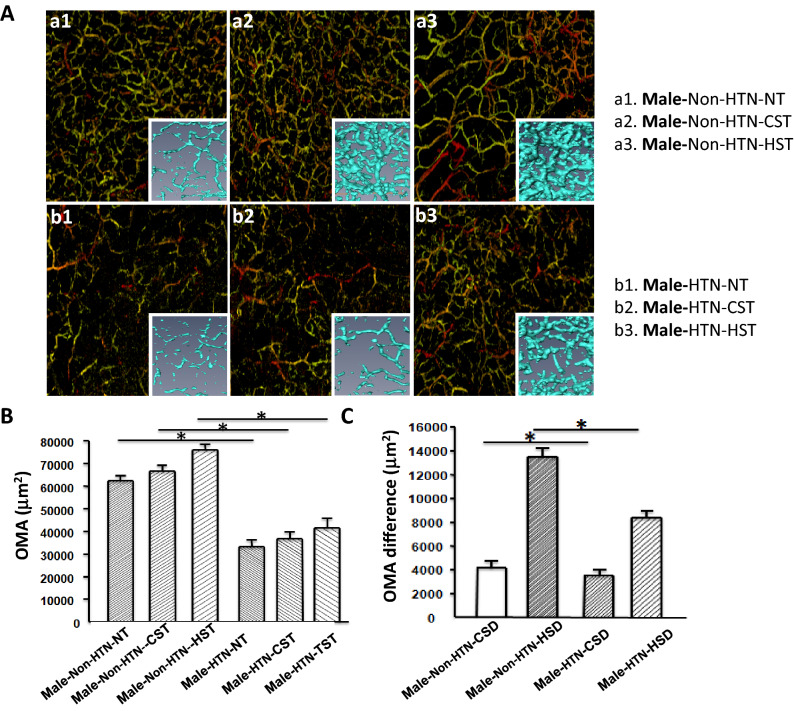
(**A**) Microvascular network imaging under the back of the hand, three-dimensional blood vessel model imaging, a1 NT, a2 CST, a3 HST in non-HTN, b1 NT in male HTN, b2 CST in male HTN, b3 HST in male HTN; (**B**) Variance histogram of blood vessel area; (**C**) Difference of blood vessel area Variance histogram; non-HTN n = 18, HTN n = 25, *mean NT, CST, HST, CSD, HSD in non-HTN and NT, CST, HST, CSD, HSD in HTN, *p* < 0.01.

**Figure 4:**
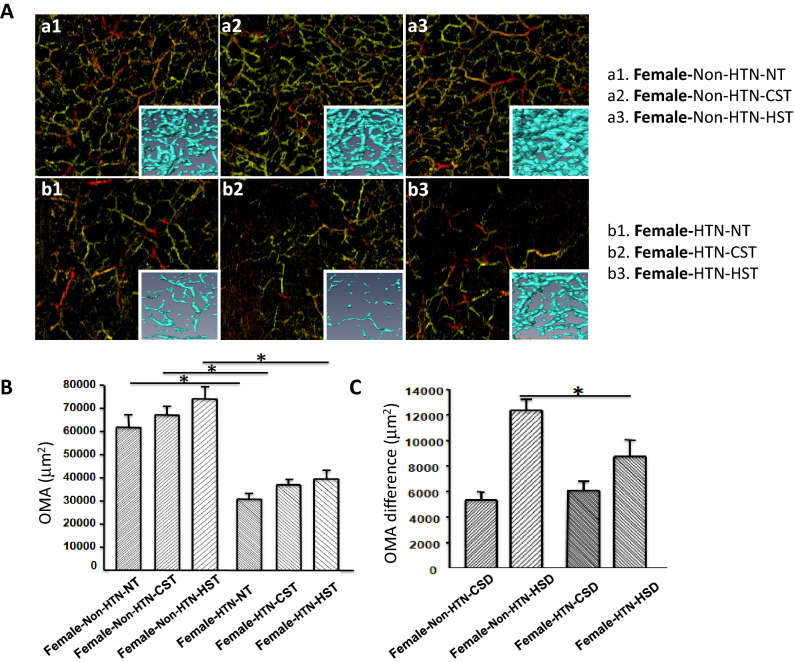
(**A**) Microvascular network imaging under the back of the hand, three-dimensional blood vessel model imaging, a1 NT, a2 CST, a3 HST in non-HTN, b1 NT in female HTN, b2 CST in female HTN, b3 HST in female HTN; (**B**) Variance histogram of blood vessel area; (**C**) Difference of blood vessel area Variance histogram; non-HTN n = 18, HTN n = 25, *mean NT, CST, HST, CSD, HSD in non-HTN and NT, CST, HST, CSD, HSD in HTN, *p* < 0.01.

The examiners were separated into male (n = 43) and female (n = 47) groups and OMA and their responses to cold and warm-stimulation were analyzed. As shown in Figs. 3 and 4, OMA was increased by cold and warm stimulation in both groups. Furthermore, OMA and their responses were significantly greater in non-HTN groups compared to those in HTN in both male and female groups (Fig. 3, 4).

Similar negative correlations were observed between baPWV or ABI and NT, CST, HST in male and female groups (Table 3). In both groups, HSD was negatively correlated with baPWV only but not with ABI (Table 3).

**Table 3:** Correlation analysis between microvessel area and arterial stiffness in males and females.

Variable	Male n = 43		Female n = 47	
	r	*p*	r	*p*
**baPWV.L**				
NT	− 0.812	0.000**	− 0.779	0.000**
CST	− 0.812	0.000**	− 0.773	0.000**
HST	− 0.814	0.000**	− 0.782	0.000**
CSD	− 0.082	0.601	0.076	0.611
HSD	− 0.548	0.000**	− 0.293	0.045*
**baPWV.R**				
NT	− 0.781	0.000**	− 0.755	0.000**
CST	− 0.776	0.000**	− 0.757	0.000**
HST	0.774	0.000**	− 0.759	0.000**
CSD	− 0.044	0.778	0.044	0.767
HSD	− 0.487	0.001*	− 0.288	0.050*
**ABI.L**				
NT	− 0.303	0.048*	− 0.556	0.000**
CST	− 0.324	0.034*	− 0.548	0.000**
HST	− 0.317	0.038*	− 0.565	0.000**
CSD	− 0.164	0.294	0.072	0.632
HSD	− 0.268	0.082	− 0.228	0.123
**ABI.R**				
NT	− 0.529	0.000**	− 0.603	0.000**
CST	− 0.537	0.000**	− 0.603	0.000**
HST	− 0.509	0.000**	− 0.570	0.000**
CSD	− 0.102	0.514	0.040	0.790
HSD	− 0.260	0.092	− 0.121	0.418

Further separation of male and female groups into non-HTN and HTN, the correlations between OMA and baPWV were absent (Tables 4, 5). These results indicate that there was no gender difference and the associations between microvascular area, their response to warm stimulation and arterial stiffness indexes are blood pressure-dependent.

**Table 4:** Correlation analysis between male microvessel area and arterial stiffness.

Variable	Non-HTN-male		HTN-male	
	r	*p*	r	*p*
**baPWV.L**				
NT	0.214	0.393	− 0.161	0.442
CST	0.046	0.857	− 0.075	0.723
HST	0.017	0.946	− 0.110	0.602
CSD	− 0.179	0.478	0.119	0.570
HSD	− 0.156	0.537	0.086	0.683
**baPWV.R**				
NT	0.071	0.778	− 0.174	0.406
CST	− 0.252	0.313	0.002	0.993
HST	0.116	0.646	− 0.098	0.642
CSD	− 0.313	0.206	0.213	0.306
HSD	0.069	0.786	0.110	0.602
**ABI.L**				
NT	0.528	0.095	0.066	0.754
CST	0.262	0.294	0.092	0.661
HST	0.386	0.114	0.042	0.842
CSD	− 0.358	0.144	0.014	0.946
HSD	− 0.056	0.826	− 0.037	0.859
**ABI.R**				
NT	0.157	0.533	− 0.267	0.198
CST	− 0.081	0.750	− 0.192	0.357
HST	0.137	0.587	− 0.018	0.933
CSD	− 0.240	0.338	0.127	0.545
HSD	0.022	0.931	0.278	0.179

**Table 5:** Correlation analysis between female microvessel area and arterial stiffness.

Variable	Non-NTN-female		HTN-female	
	r	*p*	r	*p*
**baPWV.L**				
NT	0.220	0.198	0.040	0.908
CST	0.000	1.000	0.289	0.388
HST	− 0.168	0.329	0.076	0.824
CSD	− 0.152	0.375	0.244	0.470
HSD	− 0.245	0.149	0.051	0.883
**baPWV.R**				
NT	0.042	0.807	− 0.020	0.954
CST	− 0.151	0.381	− 0.153	0.654
HST	− 0.172	0.316	− 0.007	0.983
CSD	− 0.152	0.376	0.175	0.607
HSD	− 0.158	0.358	0.005	0.987
**ABI.L**				
NT	0.192	0.262	0.083	0.807
CST	0.127	0.461	0.414	0.205
HST	− 0.055	0.750	0.333	0.318
CSD	− 0.030	0.864	0.319	0.340
HSD	− 0.142	0.408	0.279	0.407
**ABI.R**				
NT	− 0.300	0.862	0.041	0.905
CST	− 0.109	0.527	0.194	0.567
HST	− 0.024	0.890	0.370	0.263
CSD	− 0.068	0.694	0.148	0.665
HSD	− 0.003	0.984	0.343	0.302

In HTN group, average age, BMI are higher and more individuals are sedentary, with smoking and drinking habits. Therefore, we went on and analyzed the interactions between HSD and age, BMI, smoking, drinking habits, exercise or baPWV in all individuals. As shown in Table 6, Logistic Regression analysis demonstrated that only baPWV was significantly associated with HSD (OR 19.76, 95% CI, 4.959–78.733, *p* < 0.0001).

**Table 6:** Logistic Regression analysis for the association with HSD.

Variable	β	S.E	OR	95%/CL	*p*
Age (year)	− 0.023	0.024	0.978	0.933–1.025	0.349
BMI (kg/m^2^)	1.489	0.898	4.434	0.763–25.776	0.097
Smoking history	− 0.676	0.894	0.509	0.088–2.932	0.449
Drinking history	− 0.619	0.870	0.539	0.098–2.963	0.477
Exercise habits	− 0.028	0.066	0.972	0.855–1.106	0.672
baPWV.R (cm/s)	2.984	0.705	19.760	4.959–78.733	0.000*

**Figure 5:**
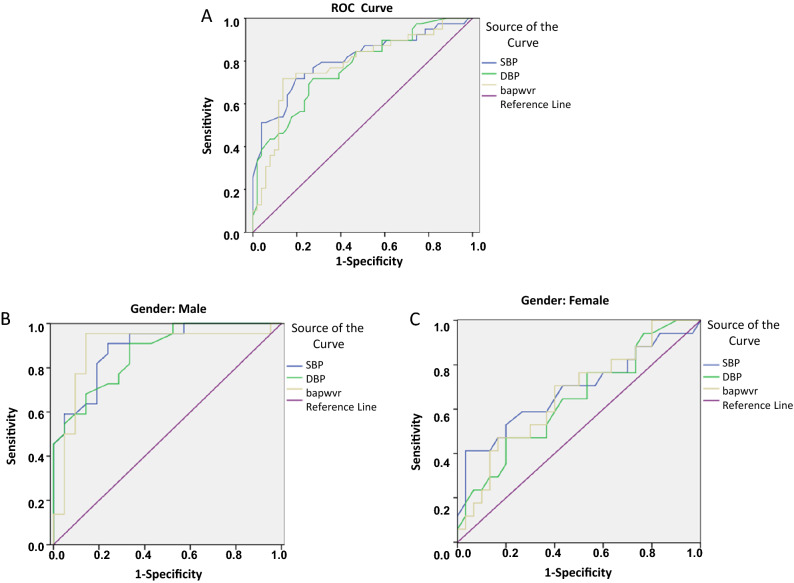
Receiver operating characteristic curves demonstrating OMA response to warm stimulation (HSD) in predicting BP and baPWV in all examiners (**A**), male group (**B**) and female group (**C**). Optimal cut off values for (**A**) is 9435.5 μm^2^, for (**B**) is 9485.5 μm^2^ (male) and for (**C**) is 7631 μm^2^ (female).

Receiver under the curve of OMA were performed to predict high blood pressure and arterial stiffness. HSD was used as an indicator to eliminate the individual discrepancy. The cut off HSD was 9435.5 μm^2^ in all 90 examiners, 9485.5 μm^2^ for male and 7631 μm^2^ for female. AUC for baPWV, SBP, DBP for all examiners were 0.781, 0.804, 0.770, respectively (*p* < 0.0001), male group were 0.887, 0.893, 0.869 (*p* < 0.0001) and female group were 0.710, 0.711, 0.645 (*p* < 0.0001, Table 7, Fig. 5). There were significant differences in SBP, DBP and baPWV between the groups at respective cut off points (*p* < 0.0001, Table 8). Higher sensitivity was observed in male group compared to those in female (Table 8).

**Table 7:** Analysis of each detection index ROC curve.

Variable	AUC	S.E	P	95%/CL	Sensitivity (%)	Specificity (%)
**All**						
baPWV (cm/s)	0.781	0.051	0.000	0.681–0.881	71.8	86.3
SBP (mmHg)	0.804	0.049	0.000	0.709–0.900	71.8	80.4
DBP (mmHg)	0.770	0.050	0.000	0.672–0.867	71.8	72.5
**Male**						
baPWV (cm/s)	0.887	0.059	0.000	0.771–1.000	95.5	85.7
SBP (mmHg)	0.893	0.047	0.000	0.800–0.986	90.9	76.2
DBP (mmHg)	0.869	0.052	0.000	0.767–0.971	90.9	66.7
**Female**						
baPWV (cm/s)	0.710	0.089	0.037	0.535–0.884	54.5	86.1
SBP (mmHg)	0.711	0.103	0.036	0.509–0.913	54.5	94.4
DBP (mmHg)	0.645	0.098	0.149	0.453–0.837	81.8	44.4

**Table 8:** Descriptive analysis between blood pressure, arterial stiffness, and microvessel area.

Variable	HSD > 9439.5	HSD ≤ 9439.5	*p*
**All**			
SBP (90–140 mmHg)	122.53 ± 16.10	147.23 ± 22.98	0.000**
DBP (60–90 mmHg)	76.14 ± 12.57	90.41 ± 14.36	0.000**
MAP (70–105 mmHg)	91.64 ± 13.13	109.23 ± 16.76	0.000**
baPWV.R (cm/s)	1247.80 ± 360.07	1699.74 ± 519.28	0.000**
baPWV.L (cm/s)	1272.27 ± 322.96	1712.33 ± 494.64	0.000**
ABI.R	1.07 ± 0.09	1.11 ± 0.11	0.038*
ABI.L	1.07 ± 0.09	1.12 ± 1.12	0.052
NT (μm^2^)	56,881.19 ± 11,803.82	41,391.35 ± 13,893.32	0.000**
CST (μm^2^)	62,153.98 ± 11,318.64	45,358.15 ± 14,286.17	0.000**
HST (μm^2^)	70,964.82 ± 12,226.68	48,498.20 ± 13,820.74	0.000**
CSD (μm^2^)	5272.78 ± 3457.05	3966.79 ± 2335.45	0.061
HSD (μm^2^)	14,083.62 ± 3458.87	7106.84 ± 1722.86	0.000**

	HSD > 9485.5	HSD ≤ 9435.5	*p*
**Male**			
SBP (90–140 mmHg)	125 ± 17.31	155.09 ± 16.81	0.000**
DBP (60–90 mmHg)	76.48 ± 13.06	95.59 ± 10.85	0.000**
MAP (70–105 mmHg)	92.71 ± 13.78	115.31 ± 12.39	0.000**
baPWV.R (cm/s)	1279.28 ± 301.22	1826.09 ± 383.32	0.000**
baPWV.L (cm/s)	1289.67 ± 303.94	1856.72 ± 349.72	0.000**
ABI.R	1.08 ± 0.12	1.14 ± 0.10	0.101
ABI.L	1.08 ± 0.11	1.12 ± 0.14	0.325
NT (μm^2^)	56,478.28 ± 12,970.27	34,809 ± 6638.57	0.000**
CST (μm^2^)	61,005.71 ± 12,179.15	37,914 ± 6695.71	0.000**
HST (μm^2^)	70,158.28 ± 13,346.6	42,360.86 ± 6510.9	0.000**
CSD (μm^2^)	4527.43 ± 2817.98	3105 ± 1647.56	0.132
HSD (μm^2^)	13,680 ± 2666.98	7551.86 ± 1602.1	0.000**

	HSD > 7631	HSD ≤ 7631	*p*
**Female**			
SBP (90–140 mmHg)	121.83 ± 15.97	142.54 ± 28.45	0.036*
DBP (60–90 mmHg)	76.63 ± 12.24	85.54 ± 18.01	0.148
MAP (70–105 mmHg)	91.72 ± 13.01	104.36 ± 20.66	0.065
baPWV.R (cm/s)	1229.67 ± 373.92	1692.72 ± 730.24	0.037*
baPWV.L (cm/s)	1259.94 ± 324.20	1670.72 ± 685.35	0.029*
ABI.R	1.06 ± 0.07	1.07 ± 0.12	0.686
ABI.L	1.06 ± 0.09	1.12 ± 0.07	0.013*
NT (μm^2^)	57,071.75 ± 11,070.85	46,252.63 ± 17,645.93	0.514
CST (μm^2^)	62,733.94 ± 11,063.03	51,379.18 ± 16,515.87	0.327
HST (μm^2^)	70,399.63 ± 11,719.45	51,907.81 ± 17,714.95	0.000**
CSD (μm^2^)	5662.19 ± 3672.65	5126.54 ± 2503.58	0.880
HSD (μm^2^)	13,327.88 ± 4293.67	5655.18 ± 1546.25	0.000**

## Discussion

MR and microvascular remodeling is associated with target organ damage in HTN patients. In the current study, we have demonstrated the associations between HTN-induced microvascular remodeling and main arterial dysfunction (arterial stiffness), which is important in early prevention of HTN-induced complications. In particular, we found that the area and the density of cutaneous microvasculature (OMA) and their responses to cold and warm stimulus, measured with recently developed OCT angiographic system, were significantly reduced in HTN group. As expected, arterial stiffness parameters (baPWV and ABI) were greater in HTN group and further analysis revealed that OMA at basal (NT), with cold and warm stimuli (CST and HST) were negatively related with baPWV and ABI in all individuals, irrespective of blood pressure group, excluding the effect of gender and suggest the importance of blood pressure in the association with OMA. Logistic Regression analysis confirmed that the differences in response to warm stimulation (HSD) were associated with baPWV, but not with age, BMI, smoking, drinking and sedentary status. In addition, ROC analysis suggest that HSD < 9435.5 μm^2^ predicts the high blood pressure and arterial stiffness. These results provide strong evidence that non-invasive OCT measurement of OMA is an efficient and sensitive technique for the assessment of HTN patients and their arterial dysfunction.

Recent development of OCT angiography provides quality analysis of the microcirculation in various tissues, including retinal, cutaneous, cerebral as well as tumor tissues^10–22^. Subcutaneous blood vessels constitute the whole microvascular network structure and the capacity for volume changes is remarkable (from 5 to 60% of cardiac output with warm stimulation^23^). Using OCT angiography, researchers from Green DJ group demonstrate that it is possible to quantify microvascular anatomic and dynamic flow rate and to make comprehensive assessment of the structure and functions of cutaneous microvasculature^24,25^. In addition, the microvascular area and diameters are increased in response to either whole-body warming (30 min of lower limb warming at 40 °C water bath)^19^ or local warming (30 min at 44 °C)^25^, further validating the measurement in human. Using similar protocols, we have found that OMA was increased following local cold (0–4 °C) and warm (42 °C) stimulation but the area and density of these parameters were significantly reduced in HTN group. These characteristics were not different between male and female, where responses were similar and OMA was reduced in HTN in both gender groups. Our results provide direct evidence of MR in peripheral cutaneous tissue of HTN patients.

It is well recognized that MR significantly reduces the tissue blood flow and increases arterial resistance. As a result, MR could be the direct cause and the consequence of BP increment. Importantly, MR is a systemic phenomenon, it provides a window to assess organ or tissue-oriented pathology through “visible” microvascular detection. Indeed, MR has been detected and confirmed in various organs, including retinal and dermal capillary using either invasive retinal angiographic (gold standards), non-invasive intravital capillaroscopy and laser Doppler flowmetry, etc.^26^. Our results provided alternative protocol of assessing MR using OCT angiography, which is convenient and efficient in healthy and HTN population. The dynamic signals indicate that the subcutaneous microvascular blood flow of healthy individual is stable but those from HTN group are disrupted with capillary thinning or compensatory hypertrophy of blood vessels. Although not studied, reduced blood flow and "ischemic" status are liable to the inflammation of target tissue, which deteriorates local blood supply, and inversely exacerbate HTN by increasing the load and reduce the distensability of the arterial walls, a vicious circle between main arteries and the microcirculation, exacerbate vascular injury^27,28^. MR and its pathology in HTN has been recognized for more than a few decades and the significance of MR in HTN remains an area of intensive research^29–33^, integrated regulations between local and systemic blood supply, ischemia, inflammation and neuronal hormonal regulation warrants further investigation.

The novel information of the current study is that OMA was reduced in HTN and OMA showed significant negative associations with baPWV and ABI in 90 participants and similar associations were observed in male or female groups, excluding gender differences. No correlations were observed in non-HTN or HTN groups because of significant differences in OMA between two groups and no associations could be observed in two clusters. Similar reductions in capillary density from dorsum of the fingers to the forearm in HTN were observed using videomicroscopy/ capillaroscopy^29,34^, confirming cutaneous MR in essential HTN. In addition, retinal capillary MR measured from OCT angiography was shown to be associated with PWV and BP in HTN^13,35^. In addition, laser Doppler Flowmetry showed functional correlations between reduced retinal capillary flow and high arterial stiffness (PWV) in HTN patients^36^. Since capillaries represent systemic microvascular network, reduced local blood flow due to MR links to respective organ dysfunction in HTN, with PVW an independent factor for HTN-induced organ damage. Of note, Logistic Regression analysis showed that only baPWV was associated with HSD changes, but not the age, BMI, smoking, drinking habit and exercise status, clearly indicating the importance of arterial stiffness on OMA parameter change in HTN. Taken together, our results demonstrate that OMA could be used to predict the pathology of the aorta and large conduit arteries. Early detection of HTN damage is important to both CV risk stratification.

Notably, we provided reference values of OMA for predicting high BP and arterial stiffness. We have analyzed HSD and CSD to avoid the bias by individual discrepancies, and we found that HSD showed significant correlations with baPWV, therefore, HSD was analyzed for predictive purposes. Our results found that the cut off values in all HTN patients were HSD < 9439.5 μm^2^, HSD < 9485.5 μm^2^ in male and HSD < 7631 μm^2^ in female groups. High sensitivity was obtained in all examiners and in male group, whereas female group showed less sensitivity, probably due to more diverse individual varieties. Nevertheless, prediction of large arterial dysfunction with microvascular area changes of each individual will be beneficial in preventing unwanted cardiovascular consequences of HTN patients. In conclusion, we have demonstrated that OMA measured from OCT angiography is reduced in HTN, indicating HTN-induced subcutaneous microvascular remodeling. Furthermore, OMA was inversely associated with arterial stiffness indexes (PWV and ABI) and HSD is a useful index to predict arterial stiffness, which is important in early prevention of HTN-induced complications. It should be stated that microcirculation measurement using OCT is not in daily clinical practice for HTN yet. However, this novel technique can be developed to observe regional dynamic blood flow, complimenting MRI or Doppler ultrasound for small vessels^37,38^. Our current study using OCT angiography proved the concept of macro- and microvascular remodeling in HTN, therefore, will significantly contribute to future clinical applications to evaluate the comorbidities of HTN patients.
